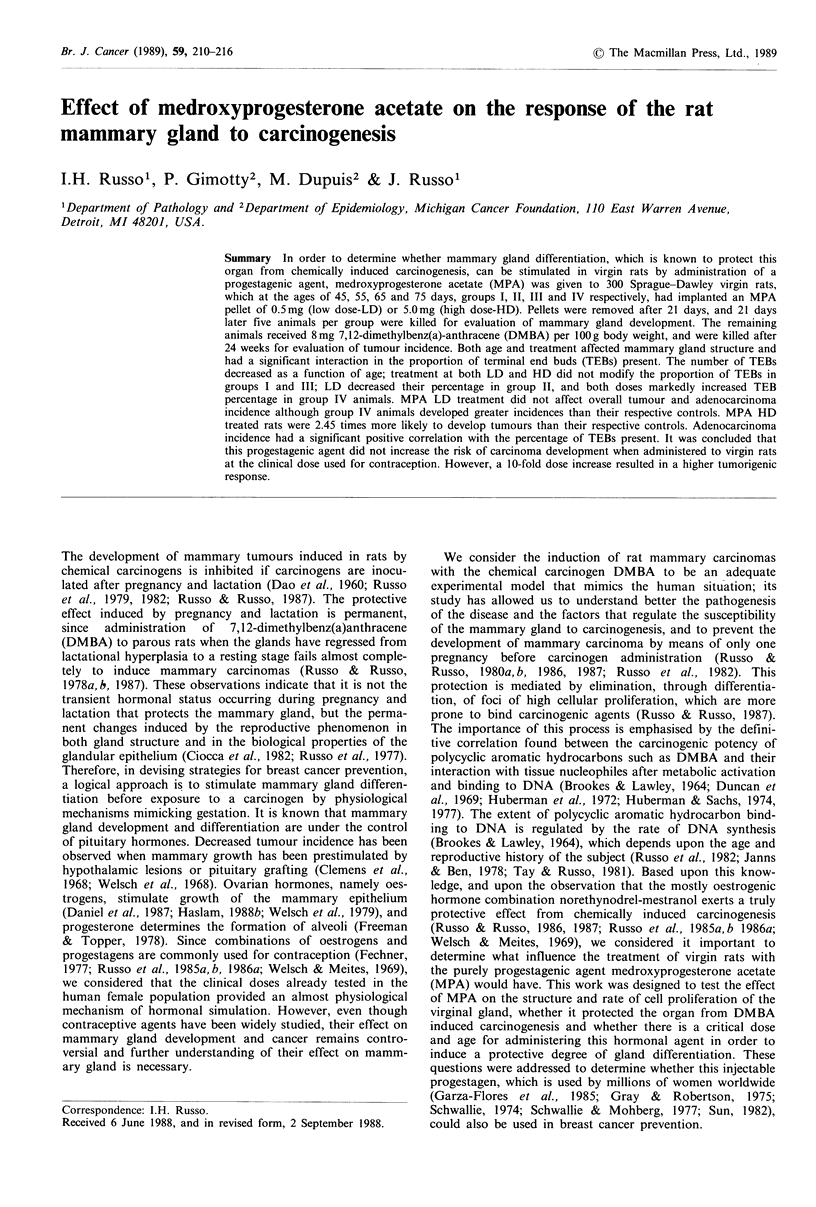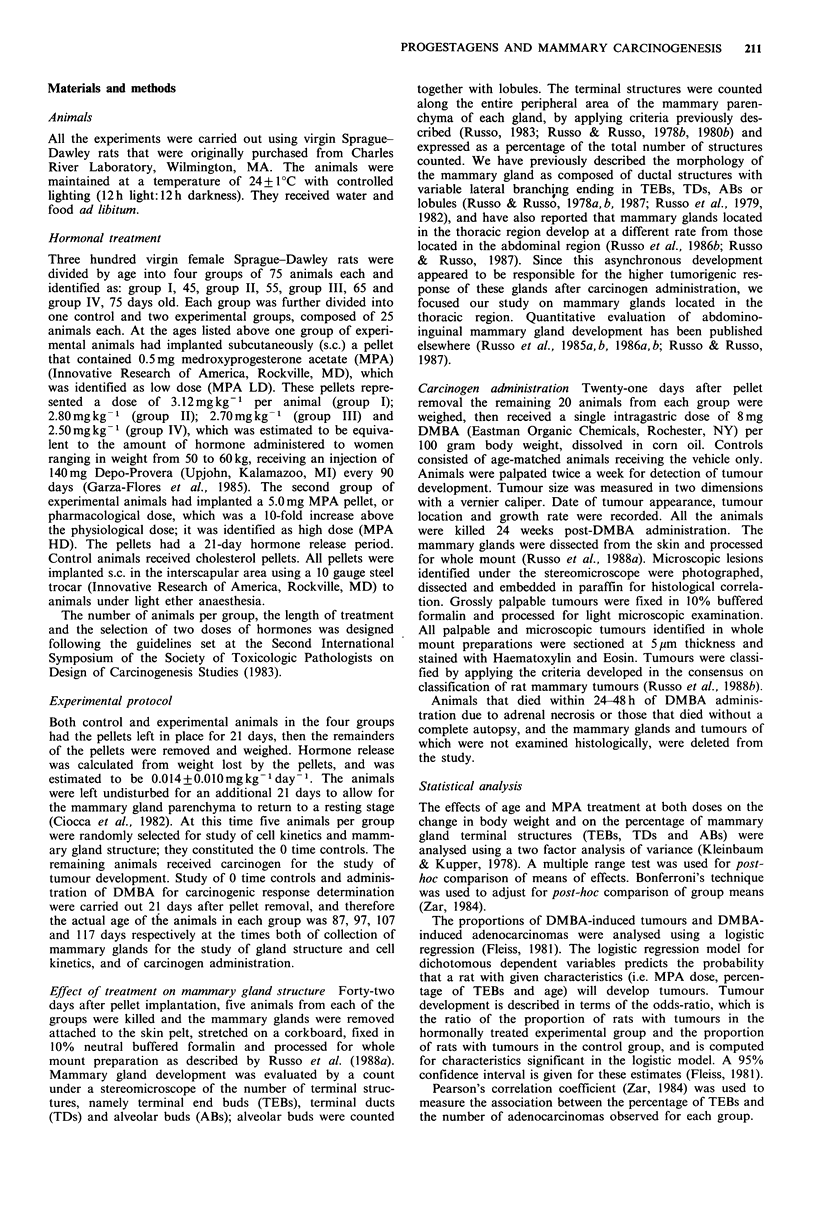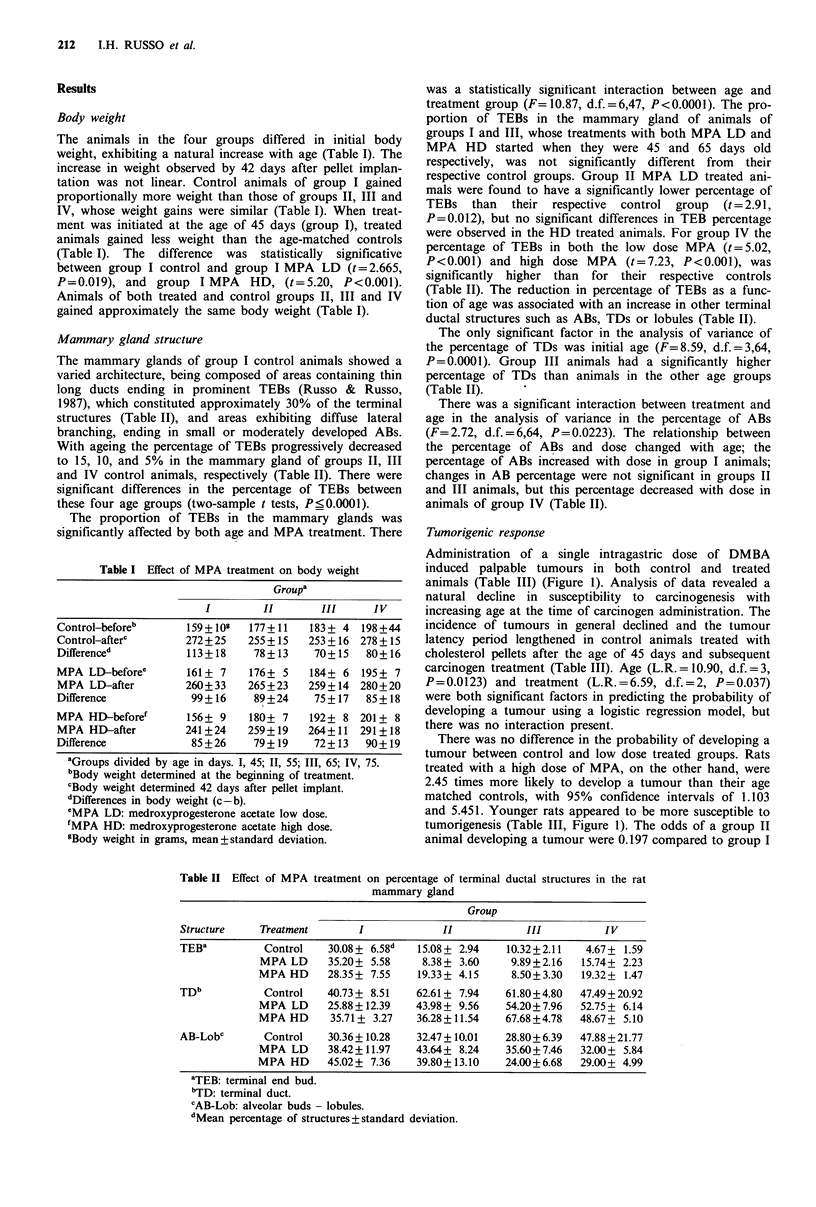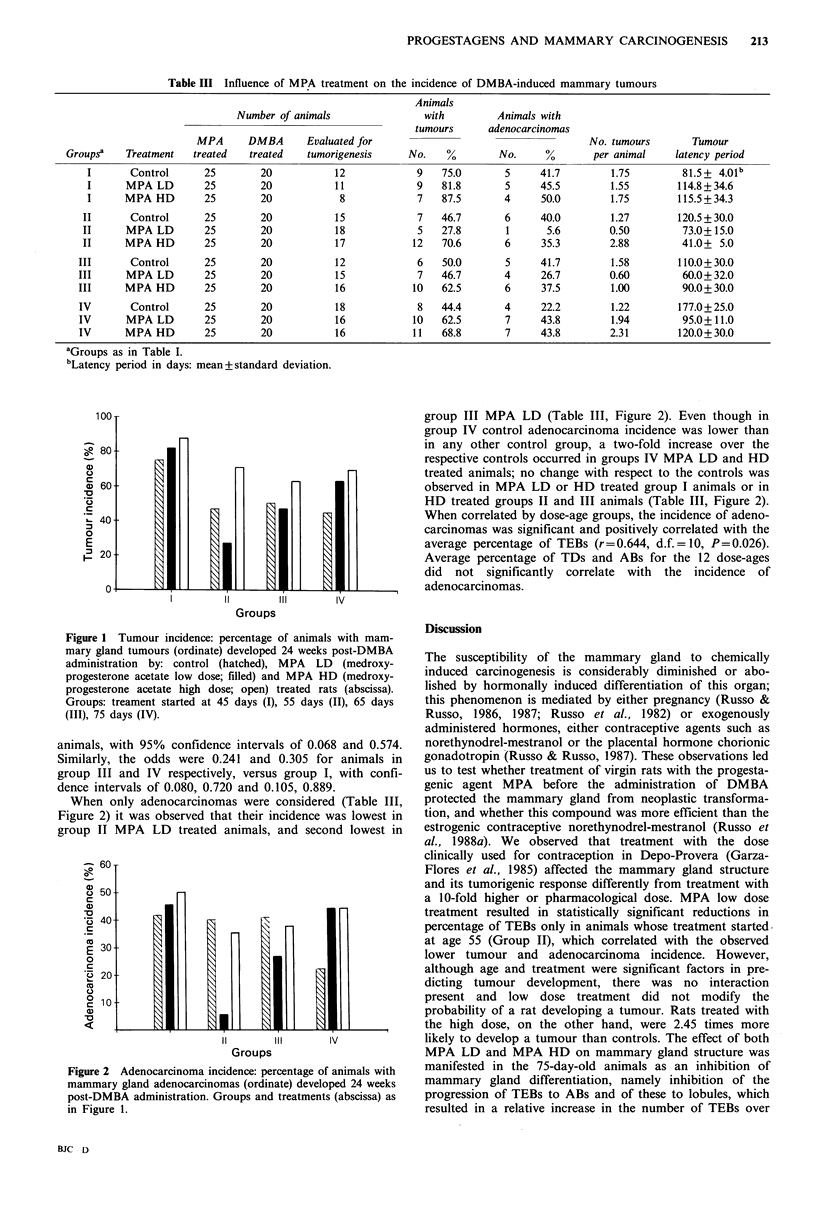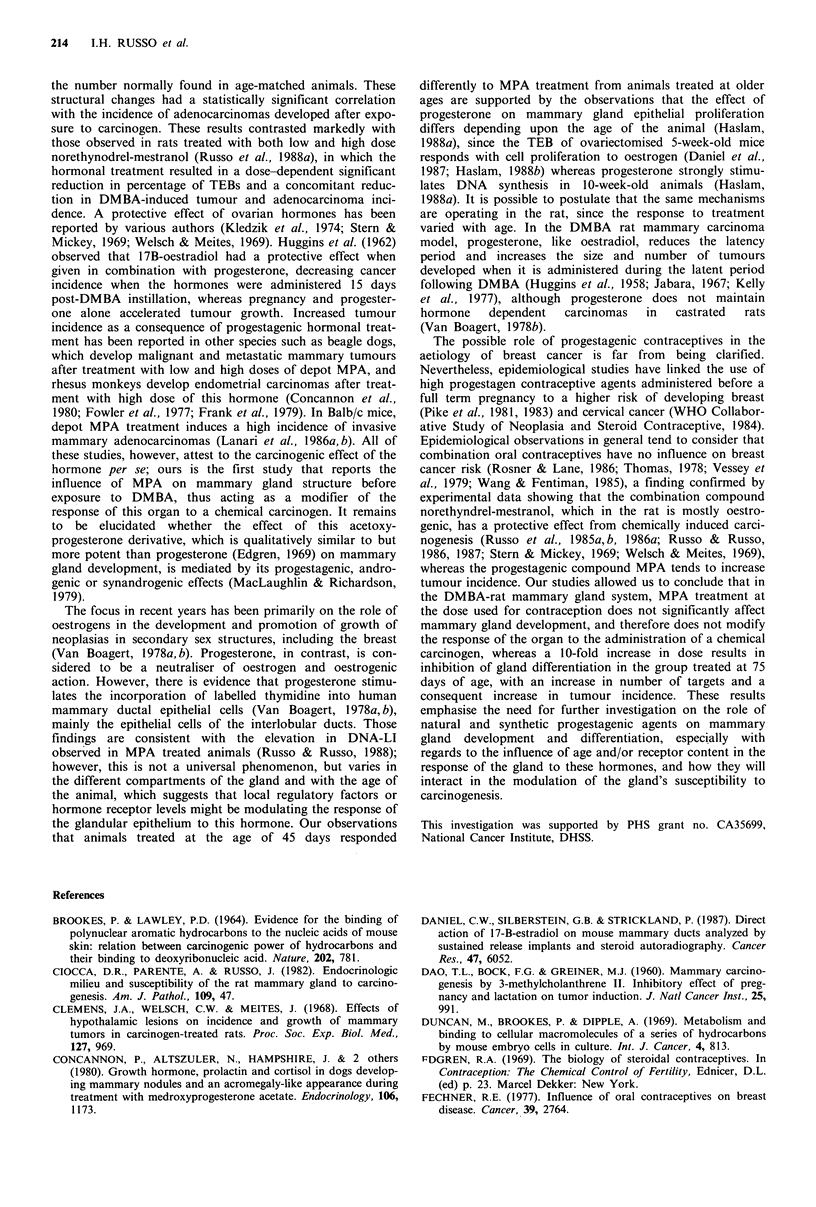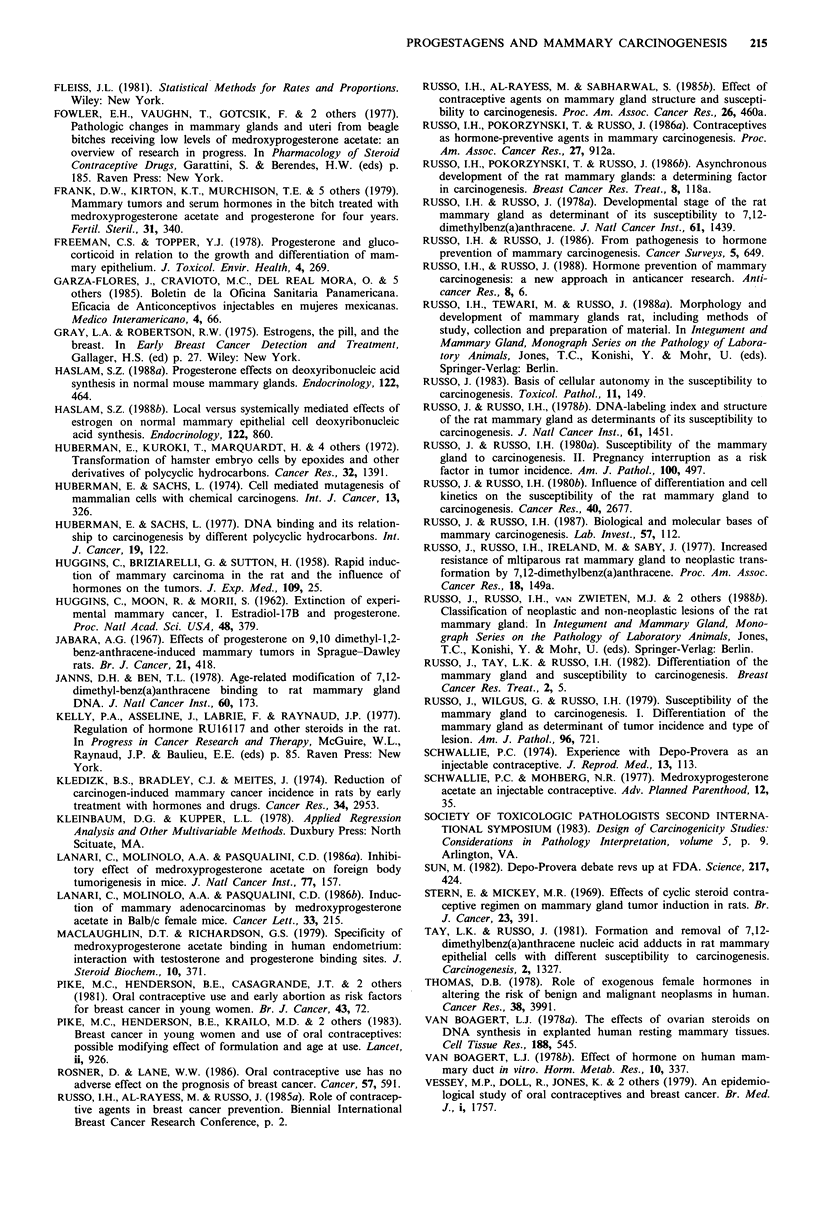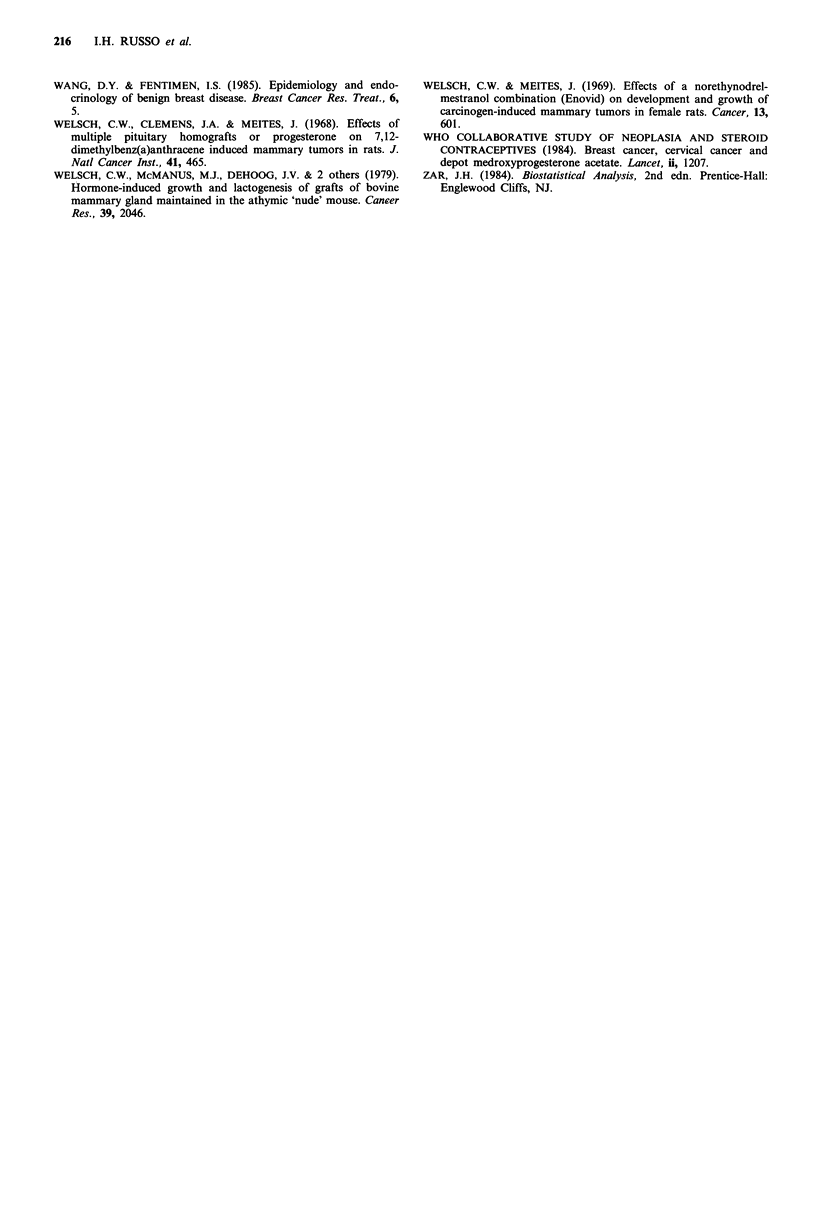# Effect of medroxyprogesterone acetate on the response of the rat mammary gland to carcinogenesis.

**DOI:** 10.1038/bjc.1989.43

**Published:** 1989-02

**Authors:** I. H. Russo, P. Gimotty, M. Dupuis, J. Russo

**Affiliations:** Department of Pathology, Michigan Cancer Foundation, Detroit 48201.

## Abstract

In order to determine whether mammary gland differentiation, which is known to protect this organ from chemically induced carcinogenesis, can be stimulated in virgin rats by administration of a progestagenic agent, medroxyprogesterone acetate (MPA) was given to 300 Sprague-Dawley virgin rats, which at the ages of 45, 55, 65 and 75 days, groups I, II, III and IV respectively, had implanted an MPA pellet of 0.5 mg (low dose-LD) or 5.0 mg (high dose-HD). Pellets were removed after 21 days, and 21 days later five animals per group were killed for evaluation of mammary gland development. The remaining animals received 8 mg 7,12-dimethylbenz(a)-anthracene (DMBA) per 100 g body weight, and were killed after 24 weeks for evaluation of tumour incidence. Both age and treatment affected mammary gland structure and had a significant interaction in the proportion of terminal end buds (TEBs) present. The number of TEBs decreased as a function of age; treatment at both LD and HD did not modify the proportion of TEBs in groups I and III; LD decreased their percentage in group II, and both doses markedly increased TEB percentage in group IV animals. MPA LD treatment did not affect overall tumour and adenocarcinoma incidence although group IV animals developed greater incidences than their respective controls. MPA HD treated rats were 2.45 times more likely to develop tumours than their respective controls. Adenocarcinoma incidence had a significant positive correlation with the percentage of TEBs present. It was concluded that this progestagenic agent did not increase the risk of carcinoma development when administered to virgin rats at the clinical dose used for contraception. However, a 10-fold dose increase resulted in a higher tumorigenic response.


*Br. J. Cancer* (1989), **59**, 210–216 © The Macmillan Press, Ltd., 1989

### Effect of medroxyprogesterone acetate on the response of the rat mammary gland to carcinogenesis

I.H. Russo[1], P. Gimotty[2], M. Dupuis[2] & J. Russo[1]

[1]*Department of Pathology and* [2]*Department of Epidemiology, Michigan Cancer Foundation, 110 East Warren Avenue, Detroit, MI 48201, USA.*

**Summary** In order to determine whether mammary gland differentiation, which is known to protect this organ from chemically induced carcinogenesis, can be stimulated in virgin rats by administration of a progestagenic agent, medroxyprogesterone acetate (MPA) was given to 300 Sprague–Dawley virgin rats, which at the ages of 45, 55, 65 and 75 days, groups I, II, III and IV respectively, had implanted an MPA pellet of 0.5 mg (low dose-LD) or 5.0 mg (high dose-HD). Pellets were removed after 21 days, and 21 days later five animals per group were killed for evaluation of mammary gland development. The remaining animals received 8 mg 7,12-dimethylbenz(a)-anthracene (DMBA) per 100 g body weight, and were killed after 24 weeks for evaluation of tumour incidence. Both age and treatment affected mammary gland structure and had a significant interaction in the proportion of terminal end buds (TEBs) present. The number of TEBs decreased as a function of age; treatment at both LD and HD did not modify the proportion of TEBs in groups I and III; LD decreased their percentage in group II, and both doses markedly increased TEB percentage in group IV animals. MPA LD treatment did not affect overall tumour and adenocarcinoma incidence although group IV animals developed greater incidences than their respective controls. MPA HD treated rats were 2.45 times more likely to develop tumours than their respective controls. Adenocarcinoma incidence had a significant positive correlation with the percentage of TEBs present. It was concluded that this progestagenic agent did not increase the risk of carcinoma development when administered to virgin rats at the clinical dose used for contraception. However, a 10-fold dose increase resulted in a higher tumorigenic response.

The development of mammary tumours induced in rats by chemical carcinogens is inhibited if carcinogens are inoculated after pregnancy and lactation (Dao *et al.*, 1960; Russo *et al.*, 1979, 1982; Russo & Russo, 1987). The protective effect induced by pregnancy and lactation is permanent, since administration of 7,12-dimethylbenz(a)anthracene (DMBA) to parous rats when the glands have regressed from lactational hyperplasia to a resting stage fails almost completely to induce mammary carcinomas (Russo & Russo, 1978*a,b*, 1987). These observations indicate that it is not the transient hormonal status occurring during pregnancy and lactation that protects the mammary gland, but the permanent changes induced by the reproductive phenomenon in both gland structure and in the biological properties of the glandular epithelium (Ciocca *et al.*, 1982; Russo *et al.*, 1977). Therefore, in devising strategies for breast cancer prevention, a logical approach is to stimulate mammary gland differentiation before exposure to a carcinogen by physiological mechanisms mimicking gestation. It is known that mammary gland development and differentiation are under the control of pituitary hormones. Decreased tumour incidence has been observed when mammary growth has been prestimulated by hypothalamic lesions or pituitary grafting (Clemens *et al.*, 1968; Welsch *et al.*, 1968). Ovarian hormones, namely oestrogens, stimulate growth of the mammary epithelium (Daniel *et al.*, 1987; Haslam, 1988*b*; Welsch *et al.*, 1979), and progesterone determines the formation of alveoli (Freeman & Topper, 1978). Since combinations of oestrogens and progestagens are commonly used for contraception (Fechner, 1977; Russo *et al.*, 1985*a,b*, 1986*a*; Welsch & Meites, 1969), we considered that the clinical doses already tested in the human female population provided an almost physiological mechanism of hormonal simulation. However, even though contraceptive agents have been widely studied, their effect on mammary gland development and cancer remains controversial and further understanding of their effect on mammary gland is necessary.

Correspondence: I.H. Russo.
Received 6 June 1988, and in revised form, 2 September 1988.

We consider the induction of rat mammary carcinomas with the chemical carcinogen DMBA to be an adequate experimental model that mimics the human situation; its study has allowed us to understand better the pathogenesis of the disease and the factors that regulate the susceptibility of the mammary gland to carcinogenesis, and to prevent the development of mammary carcinoma by means of only one pregnancy before carcinogen administration (Russo & Russo, 1980*a,b*, 1986, 1987; Russo *et al.*, 1982). This protection is mediated by elimination, through differentiation, of foci of high cellular proliferation, which are more prone to bind carcinogenic agents (Russo & Russo, 1987). The importance of this process is emphasised by the definitive correlation found between the carcinogenic potency of polycyclic aromatic hydrocarbons such as DMBA and their interaction with tissue nucleophiles after metabolic activation and binding to DNA (Brookes & Lawley, 1964; Duncan *et al.*, 1969; Huberman *et al.*, 1972; Huberman & Sachs, 1974, 1977). The extent of polycyclic aromatic hydrocarbon binding to DNA is regulated by the rate of DNA synthesis (Brookes & Lawley, 1964), which depends upon the age and reproductive history of the subject (Russo *et al.*, 1982; Janns & Ben, 1978; Tay & Russo, 1981). Based upon this knowledge, and upon the observation that the mostly oestrogenic hormone combination norethynodrel-mestranol exerts a truly protective effect from chemically induced carcinogenesis (Russo & Russo, 1986, 1987; Russo *et al.*, 1985*a,b* 1986*a*; Welsch & Meites, 1969), we considered it important to determine what influence the treatment of virgin rats with the purely progestagenic agent medroxyprogesterone acetate (MPA) would have. This work was designed to test the effect of MPA on the structure and rate of cell proliferation of the virginal gland, whether it protected the organ from DMBA induced carcinogenesis and whether there is a critical dose and age for administering this hormonal agent in order to induce a protective degree of gland differentiation. These questions were addressed to determine whether this injectable progestagen, which is used by millions of women worldwide (Garza-Flores *et al.*, 1985; Gray & Robertson, 1975; Schwallie, 1974; Schwallie & Mohberg, 1977; Sun, 1982), could also be used in breast cancer prevention.

### Materials and methods

### *Animals*

All the experiments were carried out using virgin Sprague–Dawley rats that were originally purchased from Charles River Laboratory, Wilmington, MA. The animals were maintained at a temperature of $24 \pm 1°C$ with controlled lighting (12 h light: 12 h darkness). They received water and food *ad libitum*.

### *Hormonal treatment*

Three hundred virgin female Sprague–Dawley rats were divided by age into four groups of 75 animals each and identified as: group I, 45, group II, 55, group III, 65 and group IV, 75 days old. Each group was further divided into one control and two experimental groups, composed of 25 animals each. At the ages listed above one group of experimental animals had implanted subcutaneously (s.c.) a pellet that contained 0.5 mg medroxyprogesterone acetate (MPA) (Innovative Research of America, Rockville, MD), which was identified as low dose (MPA LD). These pellets represented a dose of $3.12\,mg\,kg^{-1}$ per animal (group I); $2.80\,mg\,kg^{-1}$ (group II); $2.70\,mg\,kg^{-1}$ (group III) and $2.50\,mg\,kg^{-1}$ (group IV), which was estimated to be equivalent to the amount of hormone administered to women ranging in weight from 50 to 60 kg, receiving an injection of 140 mg Depo-Provera (Upjohn, Kalamazoo, MI) every 90 days (Garza-Flores *et al.*, 1985). The second group of experimental animals had implanted a 5.0 mg MPA pellet, or pharmacological dose, which was a 10-fold increase above the physiological dose; it was identified as high dose (MPA HD). The pellets had a 21-day hormone release period. Control animals received cholesterol pellets. All pellets were implanted s.c. in the interscapular area using a 10 gauge steel trocar (Innovative Research of America, Rockville, MD) to animals under light ether anaesthesia.

The number of animals per group, the length of treatment and the selection of two doses of hormones was designed following the guidelines set at the Second International Symposium of the Society of Toxicologic Pathologists on Design of Carcinogenesis Studies (1983).

### *Experimental protocol*

Both control and experimental animals in the four groups had the pellets left in place for 21 days, then the remainders of the pellets were removed and weighed. Hormone release was calculated from weight lost by the pellets, and was estimated to be $0.014 \pm 0.010\,mg\,kg^{-1}\,day^{-1}$. The animals were left undisturbed for an additional 21 days to allow for the mammary gland parenchyma to return to a resting stage (Ciocca *et al.*, 1982). At this time five animals per group were randomly selected for study of cell kinetics and mammary gland structure; they constituted the 0 time controls. The remaining animals received carcinogen for the study of tumour development. Study of 0 time controls and administration of DMBA for carcinogenic response determination were carried out 21 days after pellet removal, and therefore the actual age of the animals in each group was 87, 97, 107 and 117 days respectively at the times both of collection of mammary glands for the study of gland structure and cell kinetics, and of carcinogen administration.

*Effect of treatment on mammary gland structure* Forty-two days after pellet implantation, five animals from each of the groups were killed and the mammary glands were removed attached to the skin pelt, stretched on a corkboard, fixed in 10% neutral buffered formalin and processed for whole mount preparation as described by Russo *et al.* (1988*a*). Mammary gland development was evaluated by a count under a stereomicroscope of the number of terminal structures, namely terminal end buds (TEBs), terminal ducts (TDs) and alveolar buds (ABs); alveolar buds were counted together with lobules. The terminal structures were counted along the entire peripheral area of the mammary parenchyma of each gland, by applying criteria previously described (Russo, 1983; Russo & Russo, 1978*b*, 1980*b*) and expressed as a percentage of the total number of structures counted. We have previously described the morphology of the mammary gland as composed of ductal structures with variable lateral branching ending in TEBs, TDs, ABs or lobules (Russo & Russo, 1978*a,b*, 1987; Russo *et al.*, 1979, 1982), and have also reported that mammary glands located in the thoracic region develop at a different rate from those located in the abdominal region (Russo *et al.*, 1986*b*; Russo & Russo, 1987). Since this asynchronous development appeared to be responsible for the higher tumorigenic response of these glands after carcinogen administration, we focused our study on mammary glands located in the thoracic region. Quantitative evaluation of abdomino-inguinal mammary gland development has been published elsewhere (Russo *et al.*, 1985*a,b*, 1986*a,b*; Russo & Russo, 1987).

*Carcinogen administration* Twenty-one days after pellet removal the remaining 20 animals from each group were weighed, then received a single intragastric dose of 8 mg DMBA (Eastman Organic Chemicals, Rochester, NY) per 100 gram body weight, dissolved in corn oil. Controls consisted of age-matched animals receiving the vehicle only. Animals were palpated twice a week for detection of tumour development. Tumour size was measured in two dimensions with a vernier caliper. Date of tumour appearance, tumour location and growth rate were recorded. All the animals were killed 24 weeks post-DMBA administration. The mammary glands were dissected from the skin and processed for whole mount (Russo *et al.*, 1988*a*). Microscopic lesions identified under the stereomicroscope were photographed, dissected and embedded in paraffin for histological correlation. Grossly palpable tumours were fixed in 10% buffered formalin and processed for light microscopic examination. All palpable and microscopic tumours identified in whole mount preparations were sectioned at $5\,\mu m$ thickness and stained with Haematoxylin and Eosin. Tumours were classified by applying the criteria developed in the consensus on classification of rat mammary tumours (Russo *et al.*, 1988*b*).

Animals that died within 24–48 h of DMBA administration due to adrenal necrosis or those that died without a complete autopsy, and the mammary glands and tumours of which were not examined histologically, were deleted from the study.

### *Statistical analysis*

The effects of age and MPA treatment at both doses on the change in body weight and on the percentage of mammary gland terminal structures (TEBs, TDs and ABs) were analysed using a two factor analysis of variance (Kleinbaum & Kupper, 1978). A multiple range test was used for *post-hoc* comparison of means of effects. Bonferroni's technique was used to adjust for *post-hoc* comparison of group means (Zar, 1984).

The proportions of DMBA-induced tumours and DMBA-induced adenocarcinomas were analysed using a logistic regression (Fleiss, 1981). The logistic regression model for dichotomous dependent variables predicts the probability that a rat with given characteristics (i.e. MPA dose, percentage of TEBs and age) will develop tumours. Tumour development is described in terms of the odds-ratio, which is the ratio of the proportion of rats with tumours in the hormonally treated experimental group and the proportion of rats with tumours in the control group, and is computed for characteristics significant in the logistic model. A 95% confidence interval is given for these estimates (Fleiss, 1981).

Pearson's correlation coefficient (Zar, 1984) was used to measure the association between the percentage of TEBs and the number of adenocarcinomas observed for each group.

### Results

### *Body weight*

The animals in the four groups differed in initial body weight, exhibiting a natural increase with age (Table I). The increase in weight observed by 42 days after pellet implantation was not linear. Control animals of group I gained proportionally more weight than those of groups II, III and IV, whose weight gains were similar (Table I). When treatment was initiated at the age of 45 days (group I), treated animals gained less weight than the age-matched controls (Table I). The difference was statistically significative between group I control and group I MPA LD ($t=2.665$, $P=0.019$), and group I MPA HD, ($t=5.20$, $P<0.001$). Animals of both treated and control groups II, III and IV gained approximately the same body weight (Table I).

### *Mammary gland structure*

The mammary glands of group I control animals showed a varied architecture, being composed of areas containing thin long ducts ending in prominent TEBs (Russo & Russo, 1987), which constituted approximately 30% of the terminal structures (Table II), and areas exhibiting diffuse lateral branching, ending in small or moderately developed ABs. With ageing the percentage of TEBs progressively decreased to 15, 10, and 5% in the mammary gland of groups II, III and IV control animals, respectively (Table II). There were significant differences in the percentage of TEBs between these four age groups (two-sample $t$ tests, $P \leq 0.0001$).

The proportion of TEBs in the mammary glands was significantly affected by both age and MPA treatment. There was a statistically significant interaction between age and treatment group ($F=10.87$, d.f. = 6,47, $P<0.0001$). The proportion of TEBs in the mammary gland of animals of groups I and III, whose treatments with both MPA LD and MPA HD started when they were 45 and 65 days old respectively, was not significantly different from their respective control groups. Group II MPA LD treated animals were found to have a significantly lower percentage of TEBs than their respective control group ($t=2.91$, $P=0.012$), but no significant differences in TEB percentage were observed in the HD treated animals. For group IV the percentage of TEBs in both the low dose MPA ($t=5.02$, $P<0.001$) and high dose MPA ($t=7.23$, $P<0.001$), was significantly higher than for their respective controls (Table II). The reduction in percentage of TEBs as a function of age was associated with an increase in other terminal ductal structures such as ABs, TDs or lobules (Table II).

The only significant factor in the analysis of variance of the percentage of TDs was initial age ($F=8.59$, d.f. = 3,64, $P=0.0001$). Group III animals had a significantly higher percentage of TDs than animals in the other age groups (Table II).

There was a significant interaction between treatment and age in the analysis of variance in the percentage of ABs ($F=2.72$, d.f. = 6,64, $P=0.0223$). The relationship between the percentage of ABs and dose changed with age; the percentage of ABs increased with dose in group I animals; changes in AB percentage were not significant in groups II and III animals, but this percentage decreased with dose in animals of group IV (Table II).

### *Tumorigenic response*

Administration of a single intragastric dose of DMBA induced palpable tumours in both control and treated animals (Table III) (Figure 1). Analysis of data revealed a natural decline in susceptibility to carcinogenesis with increasing age at the time of carcinogen administration. The incidence of tumours in general declined and the tumour latency period lengthened in control animals treated with cholesterol pellets after the age of 45 days and subsequent carcinogen treatment (Table III). Age (L.R. = 10.90, d.f. = 3, $P=0.0123$) and treatment (L.R. = 6.59, d.f. = 2, $P=0.037$) were both significant factors in predicting the probability of developing a tumour using a logistic regression model, but there was no interaction present.

There was no difference in the probability of developing a tumour between control and low dose treated groups. Rats treated with a high dose of MPA, on the other hand, were 2.45 times more likely to develop a tumour than their age matched controls, with 95% confidence intervals of 1.103 and 5.451. Younger rats appeared to be more susceptible to tumorigenesis (Table III, Figure 1). The odds of a group II animal developing a tumour were 0.197 compared to group I

**Table I** Effect of MPA treatment on body weight

| | Group[a] | | | |
|---|---|---|---|---|
| | *I* | *II* | *III* | *IV* |
| Control–before[b] | 159±10[g] | 177±11 | 183±4 | 198±44 |
| Control–after[c] | 272±25 | 255±15 | 253±16 | 278±15 |
| Difference[d] | 113±18 | 78±13 | 70±15 | 80±16 |
| MPA LD–before[e] | 161±7 | 176±5 | 184±6 | 195±7 |
| MPA LD–after | 260±33 | 265±23 | 259±14 | 280±20 |
| Difference | 99±16 | 89±24 | 75±17 | 85±18 |
| MPA HD–before[f] | 156±9 | 180±7 | 192±8 | 201±8 |
| MPA HD–after | 241±24 | 259±19 | 264±11 | 291±18 |
| Difference | 85±26 | 79±19 | 72±13 | 90±19 |

[a]Groups divided by age in days. I, 45; II, 55; III, 65; IV, 75.
[b]Body weight determined at the beginning of treatment.
[c]Body weight determined 42 days after pellet implant.
[d]Differences in body weight (c−b).
[e]MPA LD: medroxyprogesterone acetate low dose.
[f]MPA HD: medroxyprogesterone acetate high dose.
[g]Body weight in grams, mean±standard deviation.

**Table II** Effect of MPA treatment on percentage of terminal ductal structures in the rat mammary gland

| | | Group | | | |
|---|---|---|---|---|---|
| *Structure* | *Treatment* | *I* | *II* | *III* | *IV* |
| TEB[a] | Control | 30.08±6.58[d] | 15.08±2.94 | 10.32±2.11 | 4.67±1.59 |
| | MPA LD | 35.20±5.58 | 8.38±3.60 | 9.89±2.16 | 15.74±2.23 |
| | MPA HD | 28.35±7.55 | 19.33±4.15 | 8.50±3.30 | 19.32±1.47 |
| TD[b] | Control | 40.73±8.51 | 62.61±7.94 | 61.80±4.80 | 47.49±20.92 |
| | MPA LD | 25.88±12.39 | 43.98±9.56 | 54.20±7.96 | 52.75±6.14 |
| | MPA HD | 35.71±3.27 | 36.28±11.54 | 67.68±4.78 | 48.67±5.10 |
| AB-Lob[c] | Control | 30.36±10.28 | 32.47±10.01 | 28.80±6.39 | 47.88±21.77 |
| | MPA LD | 38.42±11.97 | 43.64±8.24 | 35.60±7.46 | 32.00±5.84 |
| | MPA HD | 45.02±7.36 | 39.80±13.10 | 24.00±6.68 | 29.00±4.99 |

[a]TEB: terminal end bud.
[b]TD: terminal duct.
[c]AB-Lob: alveolar buds – lobules.
[d]Mean percentage of structures±standard deviation.

**Table III** Influence of MPA treatment on the incidence of DMBA-induced mammary tumours

| | | *Number of animals* | | | *Animals with tumours* | | *Animals with adenocarcinomas* | | | |
|---|---|---|---|---|---|---|---|---|---|---|
| *Groups*[a] | *Treatment* | *MPA treated* | *DMBA treated* | *Evaluated for tumorigenesis* | *No.* | *%* | *No.* | *%* | *No. tumours per animal* | *Tumour latency period* |
| I | Control | 25 | 20 | 12 | 9 | 75.0 | 5 | 41.7 | 1.75 | 81.5 ± 4.01[b] |
| I | MPA LD | 25 | 20 | 11 | 9 | 81.8 | 5 | 45.5 | 1.55 | 114.8 ± 34.6 |
| I | MPA HD | 25 | 20 | 8 | 7 | 87.5 | 4 | 50.0 | 1.75 | 115.5 ± 34.3 |
| II | Control | 25 | 20 | 15 | 7 | 46.7 | 6 | 40.0 | 1.27 | 120.5 ± 30.0 |
| II | MPA LD | 25 | 20 | 18 | 5 | 27.8 | 1 | 5.6 | 0.50 | 73.0 ± 15.0 |
| II | MPA HD | 25 | 20 | 17 | 12 | 70.6 | 6 | 35.3 | 2.88 | 41.0 ± 5.0 |
| III | Control | 25 | 20 | 12 | 6 | 50.0 | 5 | 41.7 | 1.58 | 110.0 ± 30.0 |
| III | MPA LD | 25 | 20 | 15 | 7 | 46.7 | 4 | 26.7 | 0.60 | 60.0 ± 32.0 |
| III | MPA HD | 25 | 20 | 16 | 10 | 62.5 | 6 | 37.5 | 1.00 | 90.0 ± 30.0 |
| IV | Control | 25 | 20 | 18 | 8 | 44.4 | 4 | 22.2 | 1.22 | 177.0 ± 25.0 |
| IV | MPA LD | 25 | 20 | 16 | 10 | 62.5 | 7 | 43.8 | 1.94 | 95.0 ± 11.0 |
| IV | MPA HD | 25 | 20 | 16 | 11 | 68.8 | 7 | 43.8 | 2.31 | 120.0 ± 30.0 |

[a]Groups as in Table I.
[b]Latency period in days: mean ± standard deviation.

**Figure 1** Tumour incidence: percentage of animals with mammary gland tumours (ordinate) developed 24 weeks post-DMBA administration by: control (hatched), MPA LD (medroxyprogesterone acetate low dose; filled) and MPA HD (medroxyprogesterone acetate high dose; open) treated rats (abscissa). Groups: treament started at 45 days (I), 55 days (II), 65 days (III), 75 days (IV).

animals, with 95% confidence intervals of 0.068 and 0.574. Similarly, the odds were 0.241 and 0.305 for animals in group III and IV respectively, versus group I, with confidence intervals of 0.080, 0.720 and 0.105, 0.889.

When only adenocarcinomas were considered (Table III, Figure 2) it was observed that their incidence was lowest in group II MPA LD treated animals, and second lowest in

**Figure 2** Adenocarcinoma incidence: percentage of animals with mammary gland adenocarcinomas (ordinate) developed 24 weeks post-DMBA administration. Groups and treatments (abscissa) as in Figure 1.

group III MPA LD (Table III, Figure 2). Even though in group IV control adenocarcinoma incidence was lower than in any other control group, a two-fold increase over the respective controls occurred in groups IV MPA LD and HD treated animals; no change with respect to the controls was observed in MPA LD or HD treated group I animals or in HD treated groups II and III animals (Table III, Figure 2). When correlated by dose-age groups, the incidence of adenocarcinomas was significant and positively correlated with the average percentage of TEBs ($r = 0.644$, d.f. = 10, $P = 0.026$). Average percentage of TDs and ABs for the 12 dose-ages did not significantly correlate with the incidence of adenocarcinomas.

### Discussion

The susceptibility of the mammary gland to chemically induced carcinogenesis is considerably diminished or abolished by hormonally induced differentiation of this organ; this phenomenon is mediated by either pregnancy (Russo & Russo, 1986, 1987; Russo *et al.*, 1982) or exogenously administered hormones, either contraceptive agents such as norethynodrel-mestranol or the placental hormone chorionic gonadotropin (Russo & Russo, 1987). These observations led us to test whether treatment of virgin rats with the progestagenic agent MPA before the administration of DMBA protected the mammary gland from neoplastic transformation, and whether this compound was more efficient than the estrogenic contraceptive norethynodrel-mestranol (Russo *et al.*, 1988*a*). We observed that treatment with the dose clinically used for contraception in Depo-Provera (Garza-Flores *et al.*, 1985) affected the mammary gland structure and its tumorigenic response differently from treatment with a 10-fold higher or pharmacological dose. MPA low dose treatment resulted in statistically significant reductions in percentage of TEBs only in animals whose treatment started at age 55 (Group II), which correlated with the observed lower tumour and adenocarcinoma incidence. However, although age and treatment were significant factors in predicting tumour development, there was no interaction present and low dose treatment did not modify the probability of a rat developing a tumour. Rats treated with the high dose, on the other hand, were 2.45 times more likely to develop a tumour than controls. The effect of both MPA LD and MPA HD on mammary gland structure was manifested in the 75-day-old animals as an inhibition of mammary gland differentiation, namely inhibition of the progression of TEBs to ABs and of these to lobules, which resulted in a relative increase in the number of TEBs over the number normally found in age-matched animals. These structural changes had a statistically significant correlation with the incidence of adenocarcinomas developed after exposure to carcinogen. These results contrasted markedly with those observed in rats treated with both low and high dose norethynodrel-mestranol (Russo *et al.*, 1988*a*), in which the hormonal treatment resulted in a dose–dependent significant reduction in percentage of TEBs and a concomitant reduction in DMBA-induced tumour and adenocarcinoma incidence. A protective effect of ovarian hormones has been reported by various authors (Kledzik *et al.*, 1974; Stern & Mickey, 1969; Welsch & Meites, 1969). Huggins *et al.* (1962) observed that 17B-oestradiol had a protective effect when given in combination with progesterone, decreasing cancer incidence when the hormones were administered 15 days post-DMBA instillation, whereas pregnancy and progesterone alone accelerated tumour growth. Increased tumour incidence as a consequence of progestagenic hormonal treatment has been reported in other species such as beagle dogs, which develop malignant and metastatic mammary tumours after treatment with low and high doses of depot MPA, and rhesus monkeys develop endometrial carcinomas after treatment with high dose of this hormone (Concannon *et al.*, 1980; Fowler *et al.*, 1977; Frank *et al.*, 1979). In Balb/c mice, depot MPA treatment induces a high incidence of invasive mammary adenocarcinomas (Lanari *et al.*, 1986*a,b*). All of these studies, however, attest to the carcinogenic effect of the hormone *per se*; ours is the first study that reports the influence of MPA on mammary gland structure before exposure to DMBA, thus acting as a modifier of the response of this organ to a chemical carcinogen. It remains to be elucidated whether the effect of this acetoxy-progesterone derivative, which is qualitatively similar to but more potent than progesterone (Edgren, 1969) on mammary gland development, is mediated by its progestagenic, androgenic or synandrogenic effects (MacLaughlin & Richardson, 1979).

The focus in recent years has been primarily on the role of oestrogens in the development and promotion of growth of neoplasias in secondary sex structures, including the breast (Van Boagert, 1978*a,b*). Progesterone, in contrast, is considered to be a neutraliser of oestrogen and oestrogenic action. However, there is evidence that progesterone stimulates the incorporation of labelled thymidine into human mammary ductal epithelial cells (Van Boagert, 1978*a,b*), mainly the epithelial cells of the interlobular ducts. Those findings are consistent with the elevation in DNA-LI observed in MPA treated animals (Russo & Russo, 1988); however, this is not a universal phenomenon, but varies in the different compartments of the gland and with the age of the animal, which suggests that local regulatory factors or hormone receptor levels might be modulating the response of the glandular epithelium to this hormone. Our observations that animals treated at the age of 45 days responded differently to MPA treatment from animals treated at older ages are supported by the observations that the effect of progesterone on mammary gland epithelial proliferation differs depending upon the age of the animal (Haslam, 1988*a*), since the TEB of ovariectomised 5-week-old mice responds with cell proliferation to oestrogen (Daniel *et al.*, 1987; Haslam, 1988*b*) whereas progesterone strongly stimulates DNA synthesis in 10-week-old animals (Haslam, 1988*a*). It is possible to postulate that the same mechanisms are operating in the rat, since the response to treatment varied with age. In the DMBA rat mammary carcinoma model, progesterone, like oestradiol, reduces the latency period and increases the size and number of tumours developed when it is administered during the latent period following DMBA (Huggins *et al.*, 1958; Jabara, 1967; Kelly *et al.*, 1977), although progesterone does not maintain hormone dependent carcinomas in castrated rats (Van Boagert, 1978*b*).

The possible role of progestagenic contraceptives in the aetiology of breast cancer is far from being clarified. Nevertheless, epidemiological studies have linked the use of high progestagen contraceptive agents administered before a full term pregnancy to a higher risk of developing breast (Pike *et al.*, 1981, 1983) and cervical cancer (WHO Collaborative Study of Neoplasia and Steroid Contraceptive, 1984). Epidemiological observations in general tend to consider that combination oral contraceptives have no influence on breast cancer risk (Rosner & Lane, 1986; Thomas, 1978; Vessey *et al.*, 1979; Wang & Fentiman, 1985), a finding confirmed by experimental data showing that the combination compound norethyndrel-mestranol, which in the rat is mostly oestrogenic, has a protective effect from chemically induced carcinogenesis (Russo *et al.*, 1985*a,b*, 1986*a*; Russo & Russo, 1986, 1987; Stern & Mickey, 1969; Welsch & Meites, 1969), whereas the progestagenic compound MPA tends to increase tumour incidence. Our studies allowed us to conclude that in the DMBA-rat mammary gland system, MPA treatment at the dose used for contraception does not significantly affect mammary gland development, and therefore does not modify the response of the organ to the administration of a chemical carcinogen, whereas a 10-fold increase in dose results in inhibition of gland differentiation in the group treated at 75 days of age, with an increase in number of targets and a consequent increase in tumour incidence. These results emphasise the need for further investigation on the role of natural and synthetic progestagenic agents on mammary gland development and differentiation, especially with regards to the influence of age and/or receptor content in the response of the gland to these hormones, and how they will interact in the modulation of the gland's susceptibility to carcinogenesis.

This investigation was supported by PHS grant no. CA35699, National Cancer Institute, DHSS.

### References

BROOKES, P. & LAWLEY, P.D. (1964). Evidence for the binding of polynuclear aromatic hydrocarbons to the nucleic acids of mouse skin: relation between carcinogenic power of hydrocarbons and their binding to deoxyribonucleic acid. *Nature*, **202**, 781.

CIOCCA, D.R., PARENTE, A. & RUSSO, J. (1982). Endocrinologic milieu and susceptibility of the rat mammary gland to carcinogenesis. *Am. J. Pathol.*, **109**, 47.

CLEMENS, J.A., WELSCH, C.W. & MEITES, J. (1968). Effects of hypothalamic lesions on incidence and growth of mammary tumors in carcinogen-treated rats. *Proc. Soc. Exp. Biol. Med.*, **127**, 969.

CONCANNON, P., ALTSZULER, N., HAMPSHIRE, J. & 2 others (1980). Growth hormone, prolactin and cortisol in dogs developing mammary nodules and an acromegaly-like appearance during treatment with medroxyprogesterone acetate. *Endocrinology*, **106**, 1173.

DANIEL, C.W., SILBERSTEIN, G.B. & STRICKLAND, P. (1987). Direct action of 17-B-estradiol on mouse mammary ducts analyzed by sustained release implants and steroid autoradiography. *Cancer Res.*, **47**, 6052.

DAO, T.L., BOCK, F.G. & GREINER, M.J. (1960). Mammary carcinogenesis by 3-methylcholanthrene II. Inhibitory effect of pregnancy and lactation on tumor induction. *J. Natl Cancer Inst.*, **25**, 991.

DUNCAN, M., BROOKES, P. & DIPPLE, A. (1969). Metabolism and binding to cellular macromolecules of a series of hydrocarbons by mouse embryo cells in culture. *Int. J. Cancer*, **4**, 813.

EDGREN, R.A. (1969). The biology of steroidal contraceptives. In *Contraception: The Chemical Control of Fertility*, Ednicer, D.L. (ed) p. 23. Marcel Dekker: New York.

FECHNER, R.E. (1977). Influence of oral contraceptives on breast disease. *Cancer*, **39**, 2764.

FLEISS, J.L. (1981). *Statistical Methods for Rates and Proportions*. Wiley: New York.

FOWLER, E.H., VAUGHN, T., GOTCSIK, F. & 2 others (1977). Pathologic changes in mammary glands and uteri from beagle bitches receiving low levels of medroxyprogesterone acetate: an overview of research in progress. In *Pharmacology of Steroid Contraceptive Drugs*, Garattini, S. & Berendes, H.W. (eds) p. 185. Raven Press: New York.

FRANK, D.W., KIRTON, K.T., MURCHISON, T.E. & 5 others (1979). Mammary tumors and serum hormones in the bitch treated with medroxyprogesterone acetate and progesterone for four years. *Fertil. Steril.*, **31**, 340.

FREEMAN, C.S. & TOPPER, Y.J. (1978). Progesterone and glucocorticoid in relation to the growth and differentiation of mammary epithelium. *J. Toxicol. Envir. Health*, **4**, 269.

GARZA-FLORES, J., CRAVIOTO, M.C., DEL REAL MORA, O. & 5 others (1985). Boletin de la Oficina Sanitaria Panamericana. Eficacia de Anticonceptivos injectables en mujeres mexicanas. *Medico Interamericano*, **4**, 66.

GRAY, L.A. & ROBERTSON, R.W. (1975). Estrogens, the pill, and the breast. In *Early Breast Cancer Detection and Treatment*, Gallager, H.S. (ed) p. 27. Wiley: New York.

HASLAM, S.Z. (1988*a*). Progesterone effects on deoxyribonucleic acid synthesis in normal mouse mammary glands. *Endocrinology*, **122**, 464.

HASLAM, S.Z. (1988*b*). Local versus systemically mediated effects of estrogen on normal mammary epithelial cell deoxyribonucleic acid synthesis. *Endocrinology*, **122**, 860.

HUBERMAN, E., KUROKI, T., MARQUARDT, H. & 4 others (1972). Transformation of hamster embryo cells by epoxides and other derivatives of polycyclic hydrocarbons. *Cancer Res.*, **32**, 1391.

HUBERMAN, E. & SACHS, L. (1974). Cell mediated mutagenesis of mammalian cells with chemical carcinogens. *Int. J. Cancer*, **13**, 326.

HUBERMAN, E. & SACHS, L. (1977). DNA binding and its relationship to carcinogenesis by different polycyclic hydrocarbons. *Int. J. Cancer*, **19**, 122.

HUGGINS, C., BRIZIARELLI, G. & SUTTON, H. (1958). Rapid induction of mammary carcinoma in the rat and the influence of hormones on the tumors. *J. Exp. Med.*, **109**, 25.

HUGGINS, C., MOON, R. & MORII, S. (1962). Extinction of experimental mammary cancer, I. Estradiol-17B and progesterone. *Proc. Natl Acad. Sci. USA*, **48**, 379.

JABARA, A.G. (1967). Effects of progesterone on 9,10 dimethyl-1,2-benz-anthracene-induced mammary tumors in Sprague–Dawley rats. *Br. J. Cancer*, **21**, 418.

JANNS, D.H. & BEN, T.L. (1978). Age-related modification of 7,12-dimethyl-benz(a)anthracene binding to rat mammary gland DNA. *J. Natl Cancer Inst.*, **60**, 173.

KELLY, P.A., ASSELINE, J., LABRIE, F. & RAYNAUD, J.P. (1977). Regulation of hormone RU16117 and other steroids in the rat. In *Progress in Cancer Research and Therapy*, McGuire, W.L., Raynaud, J.P. & Baulieu, E.E. (eds) p. 85. Raven Press: New York.

KLEDIZK, B.S., BRADLEY, C.J. & MEITES, J. (1974). Reduction of carcinogen-induced mammary cancer incidence in rats by early treatment with hormones and drugs. *Cancer Res.*, **34**, 2953.

KLEINBAUM, D.G. & KUPPER, L.L. (1978). *Applied Regression Analysis and Other Multivariable Methods*. Duxbury Press: North Scituate, MA.

LANARI, C., MOLINOLO, A.A. & PASQUALINI, C.D. (1986*a*). Inhibitory effect of medroxyprogesterone acetate on foreign body tumorigenesis in mice. *J. Natl Cancer Inst.*, **77**, 157.

LANARI, C., MOLINOLO, A.A. & PASQUALINI, C.D. (1986*b*). Induction of mammary adenocarcinomas by medroxyprogesterone acetate in Balb/c female mice. *Cancer Lett.*, **33**, 215.

MACLAUGHLIN, D.T. & RICHARDSON, G.S. (1979). Specificity of medroxyprogesterone acetate binding in human endometrium: interaction with testosterone and progesterone binding sites. *J. Steroid Biochem.*, **10**, 371.

PIKE, M.C., HENDERSON, B.E., CASAGRANDE, J.T. & 2 others (1981). Oral contraceptive use and early abortion as risk factors for breast cancer in young women. *Br. J. Cancer*, **43**, 72.

PIKE, M.C., HENDERSON, B.E., KRAILO, M.D. & 2 others (1983). Breast cancer in young women and use of oral contraceptives: possible modifying effect of formulation and age at use. *Lancet*, **ii**, 926.

ROSNER, D. & LANE, W.W. (1986). Oral contraceptive use has no adverse effect on the prognosis of breast cancer. *Cancer*, **57**, 591.

RUSSO, I.H., AL-RAYESS, M. & RUSSO, J. (1985*a*). Role of contraceptive agents in breast cancer prevention. Biennial International Breast Cancer Research Conference, p. 2.

RUSSO, I.H., AL-RAYESS, M. & SABHARWAL, S. (1985*b*). Effect of contraceptive agents on mammary gland structure and susceptibility to carcinogenesis. *Proc. Am. Assoc. Cancer Res.*, **26**, 460a.

RUSSO, I.H., POKORZYNSKI, T. & RUSSO, J. (1986*a*). Contraceptives as hormone-preventive agents in mammary carcinogenesis. *Proc. Am. Assoc. Cancer Res.*, **27**, 912a.

RUSSO, I.H., POKORZYNSKI, T. & RUSSO, J. (1986*b*). Asynchronous development of the rat mammary glands: a determining factor in carcinogenesis. *Breast Cancer Res. Treat.*, **8**, 118a.

RUSSO, I.H. & RUSSO, J. (1978*a*). Developmental stage of the rat mammary gland as determinant of its susceptibility to 7,12-dimethylbenz(a)anthracene. *J. Natl Cancer Inst.*, **61**, 1439.

RUSSO, I.H. & RUSSO, J. (1986). From pathogenesis to hormone prevention of mammary carcinogenesis. *Cancer Surveys*, **5**, 649.

RUSSO, I.H., & RUSSO, J. (1988). Hormone prevention of mammary carcinogenesis: a new approach in anticancer research. *Anticancer Res.*, **8**, 6.

RUSSO, I.H., TEWARI, M. & RUSSO, J. (1988*a*). Morphology and development of mammary glands rat, including methods of study, collection and preparation of material. In *Integument and Mammary Gland, Monograph Series on the Pathology of Laboratory Animals*, Jones, T.C., Konishi, Y. & Mohr, U. (eds). Springer-Verlag: Berlin.

RUSSO, J. (1983). Basis of cellular autonomy in the susceptibility to carcinogenesis. *Toxicol. Pathol.*, **11**, 149.

RUSSO, J. & RUSSO, I.H., (1978*b*). DNA-labeling index and structure of the rat mammary gland as determinants of its susceptibility to carcinogenesis. *J. Natl Cancer Inst.*, **61**, 1451.

RUSSO, J. & RUSSO, I.H. (1980*a*). Susceptibility of the mammary gland to carcinogenesis. II. Pregnancy interruption as a risk factor in tumor incidence. *Am. J. Pathol.*, **100**, 497.

RUSSO, J. & RUSSO, I.H. (1980*b*). Influence of differentiation and cell kinetics on the susceptibility of the rat mammary gland to carcinogenesis. *Cancer Res.*, **40**, 2677.

RUSSO, J. & RUSSO, I.H. (1987). Biological and molecular bases of mammary carcinogenesis. *Lab. Invest.*, **57**, 112.

RUSSO, J., RUSSO, I.H., IRELAND, M. & SABY, J. (1977). Increased resistance of mltiparous rat mammary gland to neoplastic transformation by 7,12-dimethylbenz(a)anthracene. *Proc. Am. Assoc. Cancer Res.*, **18**, 149a.

RUSSO, J., RUSSO, I.H., VAN ZWIETEN, M.J. & 2 others (1988*b*). Classification of neoplastic and non-neoplastic lesions of the rat mammary gland. In *Integument and Mammary Gland, Monograph Series on the Pathology of Laboratory Animals*, Jones, T.C., Konishi, Y. & Mohr, U. (eds). Springer-Verlag: Berlin.

RUSSO, J., TAY, L.K. & RUSSO, I.H. (1982). Differentiation of the mammary gland and susceptibility to carcinogenesis. *Breast Cancer Res. Treat.*, **2**, 5.

RUSSO, J., WILGUS, G. & RUSSO, I.H. (1979). Susceptibility of the mammary gland to carcinogenesis. I. Differentiation of the mammary gland as determinant of tumor incidence and type of lesion. *Am. J. Pathol.*, **96**, 721.

SCHWALLIE, P.C. (1974). Experience with Depo-Provera as an injectable contraceptive. *J. Reprod. Med.*, **13**, 113.

SCHWALLIE, P.C. & MOHBERG, N.R. (1977). Medroxyprogesterone acetate an injectable contraceptive. *Adv. Planned Parenthood*, **12**, 35.

SOCIETY OF TOXICOLOGIC PATHOLOGISTS SECOND INTERNATIONAL SYMPOSIUM (1983). *Design of Carcinogenicity Studies: Considerations in Pathology Interpretation, volume 5*, p. 9. Arlington, VA.

SUN, M. (1982). Depo-Provera debate revs up at FDA. *Science*, **217**, 424.

STERN, E. & MICKEY, M.R. (1969). Effects of cyclic steroid contraceptive regimen on mammary gland tumor induction in rats. *Br. J. Cancer*, **23**, 391.

TAY, L.K. & RUSSO, J. (1981). Formation and removal of 7,12-dimethylbenz(a)anthracene nucleic acid adducts in rat mammary epithelial cells with different susceptibility to carcinogenesis. *Carcinogenesis*, **2**, 1327.

THOMAS, D.B. (1978). Role of exogenous female hormones in altering the risk of benign and malignant neoplasms in human. *Cancer Res.*, **38**, 3991.

VAN BOAGERT, L.J. (1978*a*). The effects of ovarian steroids on DNA synthesis in explanted human resting mammary tissues. *Cell Tissue Res.*, **188**, 545.

VAN BOAGERT, L.J. (1978*b*). Effect of hormone on human mammary duct *in vitro*. *Horm. Metab. Res.*, **10**, 337.

VESSEY, M.P., DOLL, R., JONES, K. & 2 others (1979). An epidemiological study of oral contraceptives and breast cancer. *Br. Med. J.*, **i**, 1757.

WANG, D.Y. & FENTIMEN, I.S. (1985). Epidemiology and endocrinology of benign breast disease. *Breast Cancer Res. Treat.*, **6**, 5.

WELSCH, C.W., CLEMENS, J.A. & MEITES, J. (1968). Effects of multiple pituitary homografts or progesterone on 7,12-dimethylbenz(a)anthracene induced mammary tumors in rats. *J. Natl Cancer Inst.*, **41**, 465.

WELSCH, C.W., McMANUS, M.J., DEHOOG, J.V. & 2 others (1979). Hormone-induced growth and lactogenesis of grafts of bovine mammary gland maintained in the athymic 'nude' mouse. *Caneer Res.*, **39**, 2046.

WELSCH, C.W. & MEITES, J. (1969). Effects of a norethynodrel-mestranol combination (Enovid) on development and growth of carcinogen-induced mammary tumors in female rats. *Cancer*, **13**, 601.

WHO COLLABORATIVE STUDY OF NEOPLASIA AND STEROID CONTRACEPTIVES (1984). Breast cancer, cervical cancer and depot medroxyprogesterone acetate. *Lancet*, **ii**, 1207.

ZAR, J.H. (1984). *Biostatistical Analysis*, 2nd edn. Prentice-Hall: Englewood Cliffs, NJ.